# Variability of Silver Fir Needle (*Abies alba* Mill.) Anatomical Features in the Southeast Europe Natural Populations

**DOI:** 10.3390/plants13101307

**Published:** 2024-05-09

**Authors:** Vladan Popović, Aleksandar Lučić, Ljubinko Rakonjac, Aleksandar Vemić, Sanja Jovanović, Biljana M. Nikolić, Danijela Miljković

**Affiliations:** 1 Department of Genetics, Plant Breeding, Seed and Nursery Production, Institute of Forestry, Kneza Višeslava Street 3, 11030 Belgrade, Serbiasmikitis2@gmail.com (B.M.N.); 2 Department of Forest Establishment, Silviculture and Ecology, Institute of Forestry, Kneza Višeslava Street 3, 11030 Belgrade, Serbia; 3 Department of Forest Protection, Institute of Forestry, Kneza Višeslava Street 3, 11030 Belgrade, Serbia; 4 Institute for Biological Research “Siniša Stanković”, National Institute of the Republic of Serbia, University of Belgrade, 11060 Belgrade, Serbia; danijela.miljkovic@ibiss.bg.ac.rs

**Keywords:** environmental conditions, correlation anatomical needle traits, needle anatomy, populations

## Abstract

The survival of marginal/peripheral silver fir (*Abies alba* Mill.) populations in the broader region of Southeast Europe is endangered due to climate change and population decline. This study aimed to determine the level and pattern of variability for the anatomical traits of needles and the possibility of linking the pattern of phenotypic variability with environmental factors. In most of the analyzed needle traits, the statistically significant variability between populations was determined. According to the results of the multivariate principal component analysis, it is evident that the populations are distinct from each other, in three groups. The climatic factors Hargreaves reference evaporation, mean annual temperature, and growing degree-days, were statistically significantly correlated. The altitude and heating degree-days are statistically significantly correlated with the following three environmental factors: Hargreaves reference evaporation, mean annual temperature and growing degree-days, but negatively with others. The paper’s findings indicate significant moderate and high correlations between the anatomical traits of the needles’ central bundle diameter with the resin duct diameter, the distance between the vascular bundle and the resin duct and the epidermis thickness with cuticle, the resin duct diameter with the distance between the vascular bundle and the resin duct and the epidermis thickness with cuticle, as well as the distance between the vascular bundle and the resin duct with the hypodermis height and the epidermis thickness with cuticle. The results of agglomerative hierarchical clustering analysis, performed for anatomical and climatic traits, confirmed the existence of three groups of tested populations according to the altitude gradient. Research results provide knowledge on the diversity and structure of *Abies alba* populations of Southeast Europe, important for further research and guidelines for the species’ conservation and genetic variability preservation in the southern marginal distribution area and keeping in line with climate change projections.

## 1. Introduction

The decline in the number of silver fir (*Abies alba* Mill.) individuals is recorded throughout its distribution area [1], resulting from pollution, soil acidity, and rapid climate change [2,3,4,5,6]. With droughts and high temperatures in recent decades, genetic variability is one of the most important factors that can contribute to the species’ adaptation [7], and it plays a crucial role in maintaining the entire forest ecosystem [8]. Degradation of forests leads to a reduction in the size of populations, which further affects the reduction of genetic variability in the next generation, and this has a significant impact on the condition and sustainability of forest ecosystems [5,9]. The genetic variability level in these populations results from evolutionary processes, including how individual species respond to environmental changes [10]. Over the past two centuries, the silver fir forests have experienced significant reduction across their primary habitats, ranging from Poland (52° N) in the north to the northern border of Greece (40° N) in the south, and from the western Alps (5° E) in the west to Romania and Bulgaria (27° E) in the east. These forests are typically found at elevations between 500 and 800 m above sea level, with elevations increasing as one moves from north to south (over 1800 m above sea level) [11].

Numerous studies have been conducted to examine the variability of silver fir traits at different levels, such as morphological, physiological, biochemical, and genetic variability [12]. These studies have included isozyme studies [10,12,13], chloroplast microsatellite DNA [12,14,15,16], and mitochondrial DNA analyses [12,17,18,19,20]. Additionally, research has confirmed the presence of genetic variability of needle morphological traits within populations [5] and has also highlighted the genetic potential of spatially separated populations [21]. Knowledge of the phenotypic variability of silver fir is necessary due to the decline of these forests being due to the species’ sensitivity to temperature changes, lack of water, and air pollution [22].

Foliar organs, which perform photosynthesis, transpiration, and respiration, are the source of primary nutrients for plant growth [23]. The morphological and anatomical characteristics of plants are essential for their survival and resistance to uncertain and dynamic environmental conditions. Unlike reproductive organs, leaves exhibit a wide range of variations in size, structure, and shape. Both genetic and environmental factors play a role in determining the growth and differentiation of leaves. The shape of leaves is dependent on the prevailing environmental conditions, including nutrient and water availability, as well as exposure to sunlight [24]. This reflects the plant’s strategy for utilizing available resources [25]. Studies have linked leaf traits to forest ecosystem productivity [26,27] and biomass yield [28,29]. Due to their sensitivity to environmental conditions, leaves’ anatomical traits can adapt to them, making the analysis of these parameters a simple, informative, and accessible tool for examining species variability [30,31,32]. Needle analysis is also the simplest way to determine the health status of trees [33,34,35].

It is known that the variability of needle anatomy is influenced by the climatic characteristics present at different elevations. It is believed that the traits of needles responsible for photosynthesis are adapted to the specific conditions where their populations are located.

The objective of this research was to assess the variability of needle anatomy traits within and among 16 natural populations of silver fir. This study was conducted in an elevation diversity gradient ranging from 720 m above sea level to 1860 m above sea level in a part of the Southeast Europe marginal area of the silver fir population distribution.

The main objectives of this research study were to obtain answers to the following questions: Are we able to distinguish silver fir populations using anatomical traits in needles? How big are those differences? Are they related somehow to altitude or climate? The presence of genetic variability within populations and variability of needle anatomy among populations reflects adaptation to specific climatic conditions. These studies represent the first comprehensive examination of needle anatomy traits in *A. alba* populations in Southeast Europe, both at the individual and population levels.

## 2. Materials and Methods

### 2.1. Plant Material and Needle Traits

This study involved analyzing samples collected from 16 different silver fir populations in Southeast Europe. These populations were geographically distant from each other. For each population, 20 trees were selected, each of which was approximately 80–100 years old. The samples (branches with needles) were obtained from the northeast side of the canopy at a height of around 6 m, the height at which the needles were exposed to direct light in all populations. This study covered a total of 320 trees. To conduct the analysis, 10 two-year-old needles were collected randomly from each tree, resulting in a total of 3200 needles being analyzed.

The populations were situated at elevations ranging from 720 to 1860 m. This study recorded the following geographical and climatic characteristics of the populations’ localities: latitude, longitude, altitude, mean annual temperature (MAT), mean annual precipitation (MAP), and Hargreaves reference evaporation (Eref), growing degree-days above five °C (DD > 5), heating degree-days below 18 °C (DD < 18) (Table S1). Mean annual temperature and precipitation were calculated from 1961 to 2020 using the ClimateEU v4.63 software package, available at http://tinyurl.com/ClimateEU (accessed on 11 December 2023) [36].

**Figure 1:**
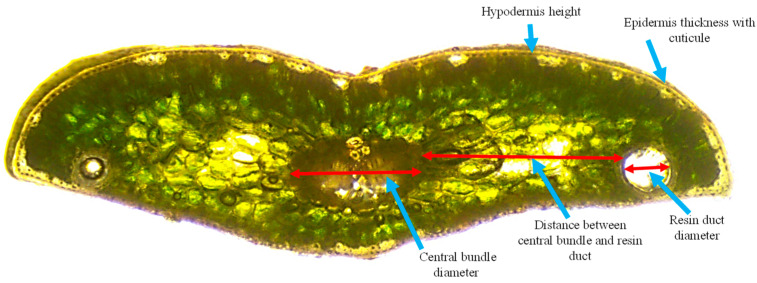
Cross-section of a silver fir needle with measured traits: central bundle diameter (CBD), resin duct diameter (RDD), the distance between central bundle and resin duct (DBDRD), hypodermis height (HH), and epidermis thickness with cuticle (ETC).

The anatomical traits of the needles (CBD—central bundle diameter, RDD—resin duct diameter, DBDRD—distance between vascular bundle and resin duct, HH—hypodermis height, ETC—epidermis thickness with cuticle) (Figure 1) were measured as the specimens were placed on microscope slides, using a light microscope (Carl Zeiss Jena, Laboval 2, Toronto Surplus, Toronto, ON, Canada) with a camera (microK) and a software package for calibration and measurement (ToupView version 3.7). The specimens (about 100 µ thick) were made manually by cutting the middle of the needle with a scalpel. Images were created as JPG files with a 2592 × 1944 pixel resolution.

### 2.2. Statistical Analyses

The descriptive analysis was performed using the MEANS procedure in SAS. The normality of data of the measured traits was analyzed by the Kolmogorov–Smirnov test (PROC UNIVARIATE, option normal), which showed the absence of a normal data distribution. Raw data were transformed by Box-Cox transformation, and an analysis of variance was performed. Analysis of variance (ANOVA) was conducted using the PROC GLM procedure to determine statistically significant differences between and within populations. The analyzed sources (factors) of variability were population (fixed factor) and tree (random factor), with the tree factor being nested within the population factor. Using Scheffé’s pairwise post hoc test, the statistical difference between the obtained mean values was tested. Different small letters indicate significant differences between populations. Pearson’s correlation analysis was conducted using the PROC CORR procedure for determining substantial relationships between the analyzed traits of silver fir needle anatomy, such as with climatic factors and between climatic factors separately. The linear regression analyses were performed (PROC REG) with climatic factors as the explanatory variable and anatomical trait response as the variable.

The principal component analysis (PCA) was a multivariate technique applied for the population variability analysis for the needle traits of each of the studied populations. For a visual presentation of the results, we used a scatterplot graph to see which needle anatomy traits contribute to separating populations.

The cluster analysis was visually presented by a dendrogram observed by the package agglomerative hierarchical clustering (AHC) (Ward’s method), performed on standardized mean values of needle traits using Euclidean distance (Ward’s method), which provided the optimal classification of the analyzed populations into homogeneous groups according to the dissimilarity.

Statistical data analysis was performed using the appropriate procedures from the software package SAS 9.1.3 (SAS Institute 2003, Cary, NC, USA) [37], for graphical presentations of XLSTAT 2014 in Microsoft Excel (https://www.xlstat.com/en/).

## 3. Results

**Figure 2:**
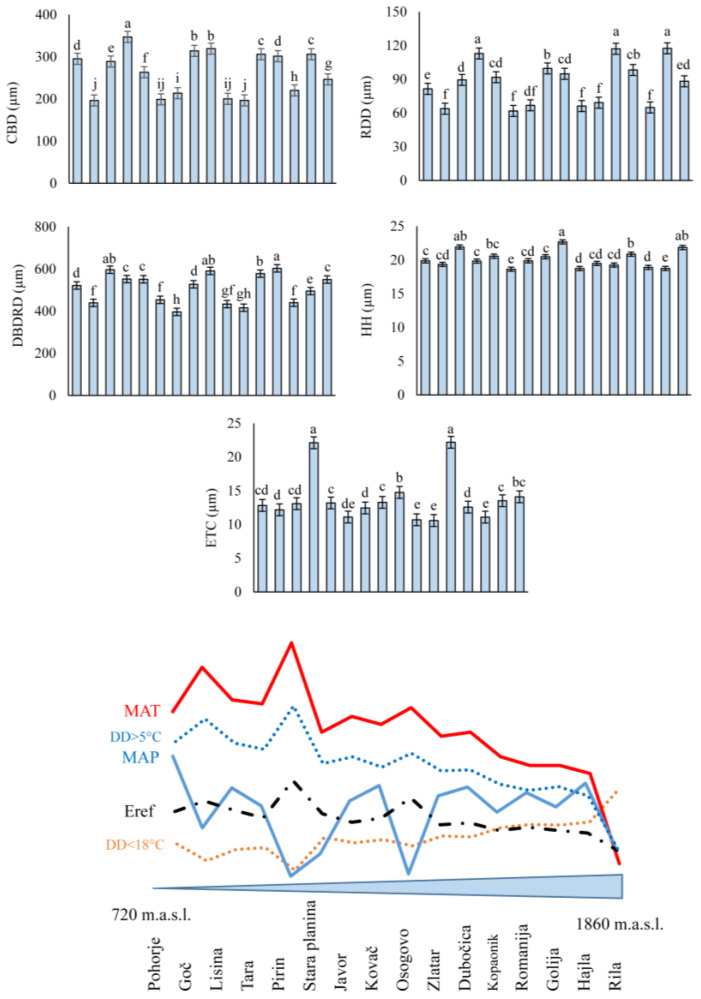
Mean values and standard errors of needles’ anatomical traits of silver fir populations (*Abies alba* Mill.) in 16 natural populations in Southeast Europe. Different small letters indicate significant differences between the populations’ mean values according to the results of Scheffé’s pairwise post hoc test. Populations are shown on the *x*-axis from the lowest (720 m.a.s.l.) to the highest elevation (1860 m.a.s.l.) and the pattern of climatic factors MAT, MAP, DD > 5, DD < 18 and Eref (abbreviations explained in Section 2).

The obtained mean values of the analyzed traits of the silver fir needle anatomy had a different pattern of variation between populations that could not be defined by the elevational diversity gradient (Figure 2). Namely, the mean values for the CBD were the lowest for the Goč population (196.40 µm) and the highest for the needles from the Tara population (347.07 µm). According to the results of Scheffé’s test, the population of Tara was statistically significantly different from the other populations.

For the RDD trait, the lowest value was for needles from Stara Planina (61.86 µm) and the highest were for the Hajla and Kopaonik populations (117.60 µm vs. 117.19 µm, respectively). Statistically, the mean values of the populations differed according to the results of Scheffé’s test.

The range of mean values for the DBDRD trait was from 396.48 µm (Javor population) to 603.53 µm (Romanija population). Statistically significant differences in mean values were confirmed by the results of Scheffé’s test.

The hypodermis height (HH) was the lowest in the needles from the Stara Planina population (18.65 µm) and the highest in the needles from the Osogovo population (22.68 µm). The mean values of the populations for the hypodermis height were statistically different according to the results of Scheffé’s test.

The mean ETC values of the silver fir needles ranged from 10.59 µm to 22.20 µm (Dubočica and Kopaonik populations, respectively). The results of Scheffé’s mean comparison test indicated statistically significant differences between the mean values.

The traits HH, RDD, and ETC had the highest values of variability parameters, coefficient of variation (CV%), in relation to the other analyzed traits CBD and DBDRD. For populations Pirin, Dubočica, Hajla, and Goč (CBD, RDD, DBDRD, HH, and ETC; respectively), the highest CV values were observed, while the lowest values were observed for the populations of Romanija, Lisina, Golija, and Tara (CBD, RDD, DBDRD, HH and ETC; respectively) (Table S2).

Mean values of the anatomical traits of silver fir needles from Southeast Europe differed among populations. Based on the results of the applied model of variance analysis for the anatomical traits of silver fir needles, all factors as a source of variability were statistically significant (all *p* < 0.0001). The obtained results confirmed statistically significant variation in all analyzed silver fir needle traits among populations (P) and within populations between trees nested in the population (Trees (P)) (Table 1).

Using the *t*-test to compare the mean values of climatic parameters between populations, it was found that all populations differ statistically significantly for each of the analyzed climatic parameters (all *p* < 0.0001). The moderate and high values of correlation parameters (Pearson coefficient) were observed for CBD with RDD, DBDRD, and ETC (0.896, 0.813, and 0.673; respectively), RDD with DBDRD (0.737) and ETC (0.744) and DBDRD with HH (0.679) and ETC (0.529).

The climatic factors Eref, MAT, and DD > 5 were statistically significantly correlated: Eref/MAT r = 0.920; DD > 5/MAT r = 0.992; Eref/DD > 5 r = 0.923, and DD < 18 r = 0.883. The altitude and DD < 18 are statistically significantly correlated with the following three environmental factors: Eref, MAT, and DD > 5, but negatively with others (altitude/Eref–r = −0.718; Altitude/MAT r = −0.853; Altitude/DD > 5 r = −0.878; DD < 18/Eref r = −0.921; DD < 18/MAT r = −0.996; DD < 18/DD > 5 r = −0.992; all *p* < 0.05). The correlation relationship between environmental factors and needle anatomy traits of silver fir was weak, with low correlation coefficient values (ranging from 0.020 to 0.196) and statistically insignificant (all *p* > 0.05). Linear regression analysis on relationships between traits was used to estimate the effect of climatic factors on the silver fir needles’ anatomical traits. Linear regression results indicate that the proportion of variance in climate traits that can be explained by anatomical traits is weak (all R^2^ < 0.02; *p* > 0.05). The fit of the model is statistically significant for all analyzed traits, which can be used as a predictor for MAP and Eref, while RDD and DBDRD can be used for altitude, DD < 5 and DD > 18 (*p* < 0.05) (Table S3).

**Figure 3:**
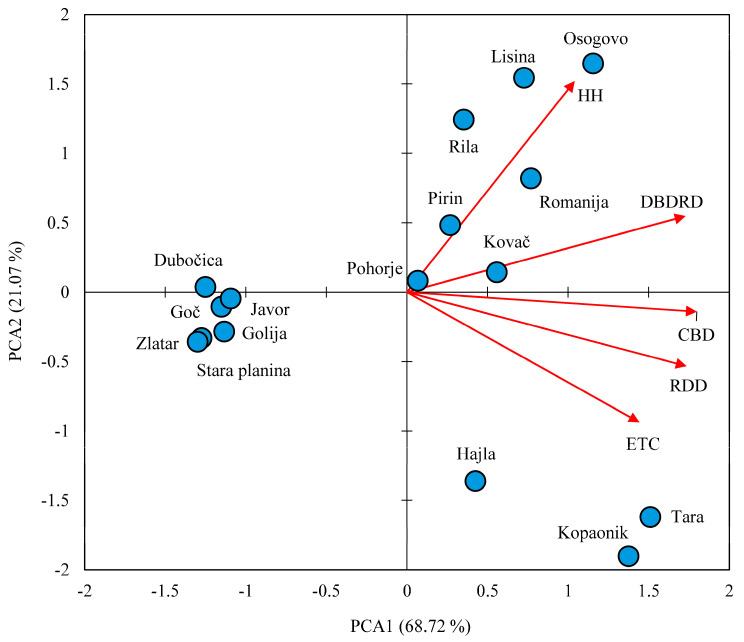
Principal component analysis (PCA) plot shows the first two primary components of the axis of population (blue circles) separation concerning the needle anatomy traits (red vectors).

The results of the multivariate analysis of the main components of the variability of silver fir needle anatomy traits and environmental factors are presented in Figure 3. According to the results of the PCA analysis, it is evident that the populations are distinct from each other and they are separated into three groups (Figure 3). The first axis of the principal components of variability accounts for 68.79% of the variability, while the second axis accounts for 21.07%, resulting in a total of 89.79%. On the scatterplot shown, the population distribution along the first axis separates Hajla, Tara, and Kopaonik (as group G1) and Dubočica, Javor, Goč, Golija, Stara Planina, and Zlatar (group G2), with smaller values of CBD, RDD, and ETC than the other populations. The third population group G3 (Osogovo, Lisina, Rila, Pirin, Romanija, Pohorje and Kovač) has higher HH and DBDRD values and is separated along the second axis from the G2 population group.

The middle factors Eref, MAT, and DD > 5, which are statistically significantly correlated with a large correlation coefficient (Eref/MAT r = 0.920; DD > 5/MAT r = 0.992; Eref/DD > 5 r = 0.923), contribute the most to the separation along the first axis, and on the other side are elevation and DD < 18 (r = 0.883). None of the environmental parameters are statistically significantly correlated with precipitation, which can be seen on the scatterplot along the length of the MAP vector, which is much shorter than the others and does not contribute to the separation of populations. Elevation and DD < 18 are statistically significantly correlated with the following three environmental factors: Eref, MAT, and DD > 5, but negatively to Altitude/Eref r = −0.718; Altitude/MAT r = −0.853; Altitude/DD > 5 r = −0.878; DD < 18/Eref r = −0.921; DD < 18/MAT r = −0.996; DD < 18/DD > 5 r = −0.992.

**Figure 4:**
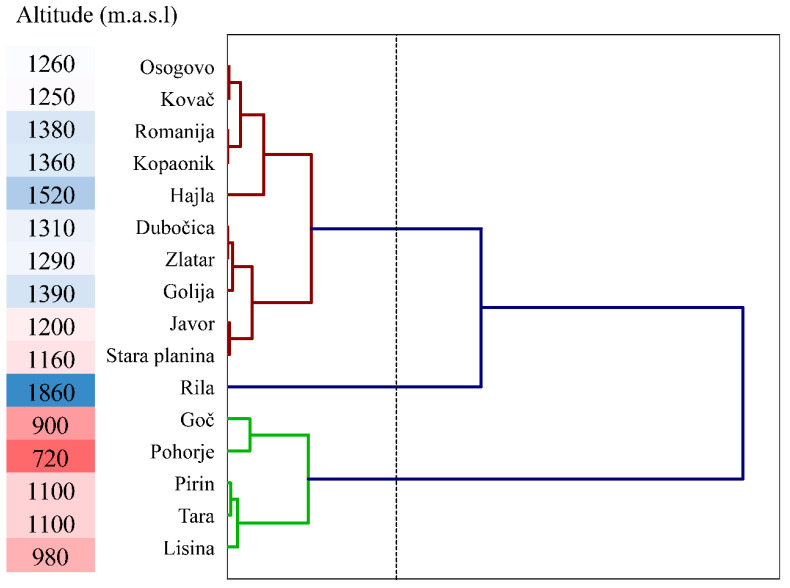
The dendrogram shows the grouping of 16 populations of silver fir (*Abies alba* Mill.) at different elevations (gradient from 720 to 1860 m.a.s.l.) based on the needle anatomy traits. The different color presents the three groups of populations (full lines) and dashed line indicates the division of the populations into 3 clusters.

Cluster analysis was performed on sixteen silver fir populations using all five needle anatomy traits. The dendrogram created using the nearest-neighbor chain algorithm for agglomerative hierarchical clustering based on Euclidean distances revealed three distinct clusters, as shown in Figure 4. The variance between clusters was found to be 68.64%, whereas within clusters, it was 31.36%. By observing the clusters with elevations (Figure 4), it can be concluded that populations located at elevations lower than 1100 m.a.s.l. formed cluster three, the Rila population located at the highest elevation of 1860 m.a.s.l. formed cluster two, while all other populations with an elevation range of 1160 m.a.s.l. up to 1520 m.a.s.l. formed the first cluster.

## 4. Discussion

Anatomy research of silver fir needles needs to be more represented in the literature. The differentiation of marginal/peripheral silver fir populations determined in our studies followed the previously described degree of species variability [13,15,18,38].

The study examined 16 populations of silver fir from various locations across Southeast Europe, which exhibit diverse physical, geographical, and climatic features. Five anatomical characteristics of silver fir needles were analyzed, including central bundle diameter, resin duct diameter, the distance between the central bundle and resin duct, hypodermis height, and epidermis thickness with cuticle. The results revealed a high degree of population and genetic variability for each characteristic. According to the results of the multivariate analysis, it is evident that the populations are distinct from each other and they are separated into three groups according to the values of silver fir needle anatomical traits.

In the preliminary studies of *A. alba* from the location of Ogorijevac (Serbia) [39], the CBD values were higher than the average values in our studies (328.49 and 263.5 µm, resp.), as well as the RDD values (139.01 and 86.5 µm., resp.), while the values for the HH (18.98 and 20.07 µm, resp.) and ETC (13.19 and 13.74 µm, resp.) were average. In [40], CBD values of *A. alba* needles from Serbia were 260.3 µm, RDD values were 89.0 µm, HH values were 20.6 µm, which are average in comparison with the presented results; ETC values were twice as high: 29.7 µm. The natural silver fir populations from North Macedonia (eight populations) have larger resin ducts [41]. The RDD ranges from 133.16 µm (population Perister) to 163.90 µm (pop. Kožuf), with an average of 141.80 µm; the HH cell (20.71 µm for the Perister population up to 25.66 µm for the Brajčinska reka population) average was 23.78 µm; and, especially, the ETC cells ranged from 29.32 µm (pop. Perister) to 38.10 µm (pop. Belasica) with an average of 35.01 [41]. The RDD in *A. alba* in Belarus (120 µm) [42] is higher than in our studies (86.5 µm). The position of *A. alba* resin ducts in our studies is medial, which is also a characteristic of *A. nephrolepis*, but not always of *A. koreana* and *A. veitchii*, where sometimes their position is marginal or submarginal [43]. In our studies, the VBD of *A. alba* averaged 263.5 µm, which is less than in *A. cilica* (479–485 µm) and *A. nordmannian* (511–594 µm) [44]. Furthermore, the RDD of *A. alba* in our studies was, on average, 86.5 µm, which is higher than in *A. cilica* (67–72, i.e., 61–66 µm) and lower than in *A. nordmannian* (137–166 µm) [44]. Also, the ETC values of *A. alba* in our studies (13.74 µm) were lower than in *A. equi-trojans* (21.5 µm), and the HH values were similar (20.07 and 20.0 µm, respectively), while the RDD values were higher (86.5 and 71.6 µm, respectively) [45].

This paper’s findings indicate significant moderate and high correlations between the anatomical traits of the needles’ CBD with RDD, the DBDRD and the ETC, the RDD with the DBDRD and the ETC, as well as the DBDRD with the HH and the ETC. Correlation of the analyses between traits and climatic or geographical variables were weak and nonsignificant. According to the linear regression analyses results (with weak goodness of fit), we found that all traits can be predictors for MAP and Eref, and for altitude, MAP, DD > 5, and DD < 18, RDD and DBDRD can be used. These findings could be a base for future study with more climatic and geographical features on sampling locations.

The traits CBD, HH, and ETC were correlated in the analysis of the anatomy of Norway spruce (*Picea abies* (L.) Karst.) needles. The climatic factors Eref, MAT, and DD > 5 were correlated with DD < 18 and altitude. This correlation pattern effectively differentiates between the populations from the Balkan Mountains and those from the Dinarides [5]. The research conducted on two conifer species (Norway spruce and silver fir) in the same environment with specific environmental conditions reveals that the species have different mechanisms of adaptation for the same environment. This finding supports the plasticity of these two species for the traits analyzed in the needle anatomy.

The results of the cluster analysis of anatomical traits indicated the formation of three distinct groups of populations with high differentiation. The first group comprised populations ranging from 1160 to 1520 m above sea level (m.a.s.l.), while the second group included populations at 1100 m.a.s.l. and below. The third group was the Rila population, located at the highest elevation of 1860 m.a.s.l. The populations of Southeast Europe can be grouped based on the anatomical traits of silver fir needles. The genetic variability was based on SSR markers analyzed in eight populations of Serbia, some of which were also studied in this paper (Stara Planina, Tara, Javor, and Zlatar i Dubočica) [15]. According to the research conducted, the studied populations exhibited a low degree of genetic diversity. The needles analyzed in this paper helped classify these populations into one sub-cluster of the first cluster, except for the Tara population, which belonged to the second cluster. The populations under observation were found at elevations ranging from 1100 m.a.s.l. to 1310 m.a.s.l.

The silver fir found in the region of Southeast Europe is just one part of the species’ vast distribution area. Evidence from fossils, paleoclimatic modeling, and genetic research suggests that coniferous trees have been present continuously during the last ice age in refuges around the Pannonian Basin [46,47,48]. The marginal/peripheral silver fir populations covered by our study are autochthonous and have survived in refugial territories under specific local environmental conditions [49]. The existing variability of silver fir is a consequence of post-glacial microevolutionary processes [21]. As environmental conditions differ along the physical, geographical, and ecological gradient of its range, the limiting factors vary in different ranges, forming specific microclimates inhabited by individuals with a fluctuating degree of sensitivity to the environment [50]. Plant populations, exposed to rapid climatic change in environmental conditions and under constant selection pressure, create specific adaptive responses along the distribution area of the species [51]. Studying the traits of plants that help them survive and reproduce in different climatic conditions can provide valuable insights into the mechanisms of adaptation and species diversity [52]. One important characteristic of plants is their ability to change their physiological and structural parts in response to varying environmental factors, known as phenotypic plasticity [53]. Changes in leaf anatomy play a significant role in adapting to new conditions, affecting plant growth and survival [54]. The survival of populations in altered environmental conditions may rely on their functional characteristics, which can be adjusted to new circumstances [52]. Tree populations are continuously impacted by the frequent occurrence of extreme climatic parameters, leading to long-term detrimental effects on forest ecosystem preservation [55,56]. The reduction in the amount of water available to plants and the increase in air temperature caused by global warming adversely impact plant species. Therefore, the adaptability of these species primarily depends on preserving genetic diversity [7,57]. According to the biogeographical distribution scale, forest endangerment is more pronounced and prevalent at the southern borders of the species’ distribution than in the center [58,59]. Environmental (among populations) and genetic (within population) variability is the basis of the response of populations to the impact of climate change. [60]. Given the issue of climate change, it has been observed that the marginal or peripheral populations are likely to be more inclined towards extreme responses to any changes in environmental factors. The Balkan Peninsula is identified as a “hot spot” in terms of climate change [61]. The populations of Balkan silver fir are located at the far southeast of its distribution range. This area is predicted to face fundamental challenges to the survival of silver fir due to the high temperatures and decreased precipitation that are expected in the future [62]. Therefore, it is essential to analyze the characteristics of silver fir needle anatomy and climate factors in order to predict how the species will respond to these changes in future climate scenarios.

The complex of environmental factors in each locality where the populations were sampled has its own specificity, and, therefore, we could not provide a precise and accurate pattern of their influence on the silver fir needle anatomy. This research on the anatomy of silver fir needles has provided valuable insights into the diversity of *A. alba* populations in Southeast Europe, specifically regarding the unique environmental factors of each analyzed population. These findings serve as a crucial foundation for future research, which should be planned and executed to establish guidelines for preserving genetic resources. Such guidelines will serve as the basis for a breeding program for this species, which will be crucial for adapting to the impacts of climate change in the region.

## Figures and Tables

**Table 1:** Results of analysis of variance (ANOVA) analysis with a population as a fixed factor (P) and the trees nested in the population (Tree (P)) as a random factor for Box-Cox transformed traits of silver fir needles (*Abies alba* Mill.) (CBD—central bundle diameter, RDD—resin duct diameter, DBDRD—distance between vascular bundle and resin duct, HH—hypodermis height, and ETC—epidermis thickness with cuticle). (df values: Population (P)–15, Tree (P)–304, Error–2880).

**Source of Variation**	**tCBD**		**tRDD**		**t DBDRD**	
	**MS**	**F**	**MS**	**F**	**MS**	**F**
Population (P)	17.32	80.41 ****	459.77	40.22 ****	18,134	26.15 ****
Tree (P)	6832.01	7.10 ****	11.43	8.58 ****	693.48	20.86 ****
Error	961.71		1.33		33.24	
**Source of Variation**	**tHH**		**tETC**			
	**MS**	**F**	**MS**	**F**		
Population (P)	14.68	8.93 ****	2.50	91.98 ****		
Tree (P)	1.64	2.31 ****	0.03	1.86 ****		
Error	0.71		0.01			

**** *p* < 0.0001.

## Data Availability

The raw data supporting the conclusions of this article will be made available by the authors on request. The data are not publicly available due to [privacy reasons].

## Supplementary Materials

The following supporting information can be downloaded at: https://www.mdpi.com/article/10.3390/plants13101307/s1, Table S1. Geographical information for sixteen *Abies alba* Mill. natural populations from Balkan: latitude, longitude, elevation and climatic parameters MAT—mean annual temperature. MAP—annual sum of precipitation, DD > 5—degree-days above 5 °C (growing degree-days), DD < 18—degree-days below 18 °C (heating degree-days), Eref—Hargreaves reference evaporation. Table S2. Descriptive statistic for silver fir needle anatomical traits: central bundle diameter (CBD), resin duct diameter (RDD), the distance between central bundle and resin duct (DBDRD), hypodermis height (HH), and epidermis thickness with cuticle (ETC) in 16 natural populations. Table S3. Results of linear regression analysis with climatic factors as explanatory variable and anatomical trait response variable.
