# Supplementary material for: Variability of Silver Fir Needle (Abies alba Mill.) Anatomical Features in the Southeast Europe Natural Populations
Source: Plants (Basel). 2024 May 9;13(10):1307. doi: 10.3390/plants13101307 (PMC11124888; doi:10.3390/plants13101307)
Supplement: Supplementary file 1 [file plants-13-01307-s001.zip › plants-2918331-supplementary.pdf]

Table S1. Geographical information for sixteen *Abies alba Mill.* natural populations from Balkan: latitude, longitude, elevation and climatic parameters MAT - mean annual temperature. MAP - annual sum of precipitation, DD > 5 – degree-days above 5 ℃ (growing degree-days), DD < 18 – degree-days below 18 ℃ (heating degree-days), Eref - Hargreaves reference evaporation.

Population	Latitude WGS84	Longitude WGS84	Elevation(m)	MAT	MAP	DD>5	DD<18	Eref
Pohorje	46.6039	15.3614	720	6.6	1251	1623	40	655
Goč	43.5603	20.7086	900	7.7	855	1801	70	688
Lisina	44.4038	17.0027	980	6.9	1076	1610	38	659
Tara	43.9094	19.4131	1100	6.8	977	1561	22	635
Pirin	41.5689	23.5583	1100	8.3	588	1903	102	753
Stara planina	43.2494	22.8380	1160	6.1	712	1449	10	648
Javor	43.4147	20.0647	1200	6.5	1004	1501	15	621
Kovač	43.5033	19.1667	1250	6.3	1089	1420	1	634
Osogovo	42.2144	22.4825	1260	6.7	601	1529	20	698
Zlatar	43.4267	19.7983	1290	6.0	1032	1392	0	613
Dubočica	43.1628	19.8775	1310	6.1	1081	1399	0	619
Kopaonik	43.2841	20.8522	1360	5.5	942	1286	0	595
Romanija	43.9297	18.4964	1380	5.3	1050	1237	0	605
Golija	43.3480	20.4472	1390	5.3	971	1266	0	595
Hajla	42.7806	20.1886	1520	5.1	1102	1193	0	587
Rila	42.1517	23.5597	1860	2.9	757	774	0	533

Table S2. Descriptive statistic for silver fir needle anatomical traits: central bundle diameter (CBD), resin duct diameter (RDD), the distance between central bundle and resin duct (DBDRD), hypodermis height (HH), and epidermis thickness with cuticle (ETC) in 16 natural populations.

	CBD					RDD				
Population	Mean	Variance	Stdev	Quartile range	CV(%)	Mean	Variance	Stdev	Quartile range	CV(%)
Pohorije	295.34	1052.03	32.43	46.82	10.98	81.53	223.33	14.94	21.55	18.33
Goč	196.40	535.22	23.13	32.16	11.78	63.87	230.75	15.19	18.84	23.78
Lisina	289.02	862.11	29.36	40.61	10.16	89.50	317.80	17.83	22.89	19.92
Tara	347.07	1574.32	39.68	48.66	11.43	112.99	474.14	21.77	29.35	19.27
Pirin	263.38	5756.61	75.87	45.48	28.81	91.84	387.48	19.68	26.11	21.43
Stara planina	199.16	542.12	23.28	32.51	11.69	61.86	252.37	15.89	19.61	25.68
Javor	213.93	900.09	30.00	47.52	14.02	66.87	257.75	16.05	22.19	24.01
Kovač	314.26	2198.85	46.89	67.79	14.92	99.70	623.65	24.97	37.95	25.05
Osogovo	319.53	2147.67	46.34	66.00	14.50	94.90	543.72	23.32	35.14	24.57
Zlatar	200.32	687.73	26.22	36.77	13.09	66.27	297.52	17.25	23.26	26.03
Dubočica	196.59	621.90	24.94	32.14	12.69	69.19	421.04	20.52	28.20	29.65
Kopaonik	306.25	1694.39	41.16	48.70	13.44	117.19	552.16	23.50	26.44	20.05
Romanija	301.75	963.40	31.04	41.07	10.29	98.33	294.75	17.17	21.69	17.46
Golija	220.41	657.94	25.65	39.89	11.64	64.95	278.97	16.70	22.61	25.72
Hajla	306.35	2741.90	52.36	69.13	17.09	117.60	549.50	23.44	26.75	19.93
Rila	246.64	1418.75	37.67	52.90	15.27	88.26	408.57	20.21	22.09	22.90

Table S2. Continued.

	DBDRD					HH				
Population	Mean	Variance	Stdev	Quartile range	CV(%)	Mean	Variance	Stdev	Quartile range	CV(%)
Pohorije	521.92	7386.86	85.95	115.22	16.47	19.88	13.70	3.70	4.76	18.62
Goč	439.37	3211.32	56.67	87.50	12.90	19.36	15.92	3.99	5.09	20.61
Lisina	596.79	2443.08	49.43	62.22	8.28	21.93	19.22	4.38	5.45	19.99
Tara	552.43	6917.14	83.17	100.76	15.06	19.85	15.87	3.98	4.52	20.07
Pirin	551.36	5317.46	72.92	94.71	13.23	20.57	17.55	4.19	5.49	20.37
Stara planina	453.82	2184.60	46.74	63.49	10.30	18.65	14.02	3.74	4.82	20.08
Javor	396.48	3129.47	55.94	73.03	14.11	19.89	17.07	4.13	5.28	20.77
Kovač	527.59	5164.05	71.86	103.81	13.62	20.49	16.56	4.07	5.60	19.87
Osogovo	591.21	9173.23	95.78	144.79	16.20	22.68	18.00	4.24	5.16	18.71
Zlatar	433.55	3512.62	59.27	80.87	13.67	18.74	12.56	3.54	4.39	18.91
Dubočica	416.27	4765.43	69.03	95.07	16.58	19.51	17.05	4.13	5.09	21.17
Kopaonik	578.26	7086.32	84.18	95.60	14.56	19.26	16.54	4.07	5.00	21.12
Romanija	603.53	7241.49	85.10	112.51	14.10	20.86	17.91	4.23	5.20	20.29
Golija	440.11	4200.07	64.81	99.30	14.73	18.92	11.40	3.38	3.84	17.85
Hajla	495.43	5954.83	77.17	112.96	15.58	18.76	21.17	4.60	5.84	24.53
Rila	550.04	6104.15	78.13	118.43	14.20	21.85	17.59	4.19	4.96	19.19

Table S2. Continued.

	ETC				
Population	Mean	Variance	Stdev	Quartile range	CV(%)
Pohorije	12.83	8.89	2.98	3.36	23.24
Goč	12.18	13.83	3.72	4.23	30.54
Lisina	13.09	8.15	2.85	3.54	21.81
Tara	22.12	16.43	4.05	5.35	18.32
Pirin	13.20	10.32	3.21	4.21	24.34
Stara planina	11.11	7.21	2.69	3.44	24.17
Javor	12.46	10.68	3.27	4.07	26.23
Kovač	13.29	9.50	3.08	4.02	23.19
Osogovo	14.76	11.00	3.32	3.87	22.46
Zlatar	10.70	7.03	2.65	3.67	24.78
Dubočica	10.59	7.89	2.81	3.38	26.53
Kopaonik	22.20	23.45	4.84	6.04	21.82
Romanija	12.59	7.86	2.80	3.69	22.28
Golija	11.10	4.99	2.23	2.84	20.14
Hajla	13.53	11.59	3.40	4.44	25.15
Rila	14.11	10.29	3.21	4.43	22.74

Table S3. Results of linear regression analysis with climatic factors as explanatory variable and anatomical trait response variable.

		Regressions coefficients				Regression statistics			
Trait		Coefficients	SE	t-value	*p*	R^2	Adj R^2	F value	*p*
CBD	Intercept	270.24	5.63	48.01	<0.0001	0.0005	0.0002	1.4	0.2236
	Altitude	-0.005	0.004	-1.22	0.2236				
RDD	Intercept	65.16	2.36	27.59	<0.0001	0.0260	0.0257	85.47	**<0.0001**
	Altitude	0.017	0.009	9.25	<0.0001				
DBDRD	Intercept	490.65	8.72	56.26	<0.0001	0.0015	0.0012	4.75	0.0295
	Altitude	0.015	0.007	2.18	0.0295				
HH	Intercept	19.51	0.37	52.65	<0.0001	0.0007	0.0004	2.39	0.1220
	Altitude	0.0004	0.0003	1.55	0.1220				
ETC	Intercept	13.01	0.412	31.54	<0.0001	0.0010	0.007	3.26	0.0712
	Altitude	0.0006	0.0003	1.80	0.0712				

		Regressions coefficients				Regression statistics			
Trait		Coefficients	SE	t-value	*p*	R^2	Adj R^2	F value	*p*
CBD	Intercept	261.65	5.99	43.67	<0.0001	0.0000	-0.0003	0.10	0.7499
	MAT	0.31	0.96	0.32	0.7499				
RDD	Intercept	100.78	2.53	39.77	<0.0001	0.0101	0.0098	32.70	**<0.0001**
	MAT	-2.32	0.40	-5.72					
DBDRD	Intercept	530.09	9.28	57.12	<0.001	0.0016	0.0013	5.23	0.0223
	MAT	-3.40	1.49	-2.29	0.0223				
HH	Intercept	20.27	0.39	51.38	<0.0001	0.0001	-0.0002	0.26	0.6121
	MAT	-0.032	0.06	-0.51	0.6121				
ETC	Intercept	14.15	0.43	32.22	<0.0001	0.0003	-0.0000	0.90	0.3427

	MAT	-0.07	0.07	-0.95	0.3427				

		Regressions coefficients				Regression statistics			
Trait		Coefficients	SE	t-value	*p*	R^2	Adj R^2	F value	*p*
CBD	Intercept	228.71	5.87	38.96	<0.0001	0.0113	0.0110	36.53	**<0.0001**
	MAP	0.04	0.006	6.04	<0.0001				
RDD	Intercept	78.77	2.51	31.4	<0.0001	0.0031	0.0028	10.03	**0.0016**
	MAP	0.008	0.003	3.17	0.0016				
DBDRD	Intercept	565.79	9.10	62.21	<0.0001	0.0124	0.0121	40.11	**<0.0001**
	MAP	0.06	0.01	-6.33	<0.0001				
HH	Intercept	23.04	0.39	59.93	<0.0001	0.0083	0.0080	26.73	**<0.0001**
	MAP	-0.002	0.0004	-5.17	<0.0001				
ETC	Intercept	17.88	0.4322	34.42	<0.0001	0.002	0.0019	7.17	**0.0074**
	MAP	-0.0012	0.0005	-2.68	0.0074				

Table S3. Continued.

		Regressions coefficients				Regression statistics			
Trait		Coefficients	SE	t-value	*p*	R^2	Adj R^2	F value	*p*
CBD	Intercept	269.14	6.47	41.61	<0.0001	0.0002	-0.0001	0.78	0.3784
	DD>5	-0.004	0.004	-0.88	0.3784				
RDD	Intercept	106.43	2.73	39.04	<0.0001	0.0169	0.0165	54.82	**<0.0001**
	DD>5	-0.014	0.0019	-7.40	<0.0001				
DBDRD	Intercept	533.86	10.01	53.29	<0.0001	0.0019	0.0016	6.22	**<0.0127**
	DD>5	-0.017	0.007	-2.49	0.0127				
HH	Intercept	20.33	0.43	47.72	<0.0001	0.0001	-0.0002	0.36	0.5490
	DD>5	-0.0002	0.0003	-0.60	0.5490				
ETC	Intercept	14.58	0.47	30.76	<0.0001	0.0010	0.0007	3.21	0.0734
	DD>5	-0.0006	0.0003	-1.79	0.0734				

		Regressions coefficients				Regression statistics			
Trait		Coefficients	SE	t-value	*p*	R^2	Adj R^2	F value	*p*
CBD	Intercept	263.87	11.81	22.35	<0.0001	0.0000	-0.0003	0.00	0.9767
	DD>18	-0.0001	0.003	-0.03	0.9767				
RDD	Intercept	57.03	4.99	11.43	<0.0001	0.0109	0.0106	35.32	**<0.0001**
	DD>18	0.007	0.001	5.94	<0.0001				
DBDRD	Intercept	464.59	1.28	25.41	<0.0001	0.0019	0.0016	6.02	**0.0142**
	DD>18	0.01	0.004	2.45	0.0142				
HH	Intercept	19.64	0.78	25.26	<0.0001	0.0001	-0.0002	0.32	0.5728
	DD>18	0.00001	0.0001	0.56	0.5728				
ETC	Intercept	12.73	0.86	17.72	<0.0001	0.0004	0.0001	1.37	0.2413
	DD>18	0.0003	0.0001	1.17	0.2413				

		Regressions coefficients				Regression statistics			

Trait		Coefficients	SE	t-value	*p*	R^2	Adj R^2	F value	*p*
CBD	Intercept	231.39	14.44	16.02	<0.0001	0.0016	0.0012	4.98	**0.0257**
	Eref	0.05	0.23	2.23	0.0257				
RDD	Intercept	115.32	6.12	18.84	<0.0001	0.0069	0.0066	22.21	**<0.0001**
	Eref	-0.05	0.010	-4.71	<0.0001				
DBDRD	Intercept	405.85	22.33	18.18	<0.0001	0.0067	0.0064	21.58	**<0.0001**
	Eref	0.16	0.04	4.65	<0.0001				
HH	Intercept	17.13	0.95	18.02	<0.0001	0.0030	0.0027	9.68	**0.0019**
	Eref	0.005	0.002	3.11	0.0019				
ETC	Intercept	17.57	1.06	16.63	<0.0001	0.0041	0.0038	13.24	**<0.0003**
	Eref	-0.006	-0.0065	-3.64	0.0003				
